# Urine Levels of Matrix Metalloproteinases and Tissue Inhibitor of Metalloproteinases in Children with Type 1 Diabetes Mellitus

**DOI:** 10.4274/jcrpe.galenos.2018.2018.0221

**Published:** 2019-05-28

**Authors:** Zeynep Yürük Yıldırım, Alev Yılmaz, Cemile Pehlivanoğlu, Asuman Gedikbaşı, Mehmet Yıldız, Ahmet Dirican, Rüveyde Bundak, Feyza Darendeliler, Sevinç Emre, Ahmet Nayır

**Affiliations:** 1 İstanbul University İstanbul Faculty of Medicine, Department of Pediatric Nephrology, İstanbul, Turkey; 2 Bakırköy Dr. Sadi Konuk Training and Research Hospital, Clinic of Biochemistry, İstanbul, Turkey; 3 İstanbul University İstanbul Faculty of Medicine, Department of Pediatrics, İstanbul, Turkey; 4 İstanbul University İstanbul Faculty of Medicine, Department of Biostatistics, İstanbul, Turkey; 5 İstanbul University Istanbul Faculty of Medicine, Department of Pediatric Endocrinology, İstanbul, Turkey

**Keywords:** Type 1 diabetes mellitus, diabetic nephropathy, children, biomarker, MMP, TIMP

## Abstract

**Objective:**

Histopathological changes in the kidney in type 1 diabetes mellitus (T1DM) begin before detection of microalbuminuria. Therefore, there is interest in finding a better biomarker for the early detection of diabetic kidney injury. The aim of this present study was to determine whether urinary indicators of fibrosis are detectable early in the development of T1DM in children and if they may predict progressive renal injury.

**Methods:**

Urinary matrix metalloproteinase 2 and 9 (MMP2 and MMP9), tissue inhibitor of metalloproteinase 1 and 2 (TIMP1 and TIMP2) and transforming growth factor-β1 (TGF-β1) were assessed in 33 patients with T1DM with normal renal functions and in 24 healthy controls. Microalbuminuria was not present in the patient group with the exception of three patients. The results were adjusted to urine creatinine (Cr) and the differences between patients and controls were evaluated. These measurements were repeated after one year and the results were compared with the first year results.

**Results:**

Urine MMP2/Cr, MMP9/Cr, TIMP1/Cr, TIMP2/Cr, TGF-β1/Cr were not different between the patient and control groups (p>0.05). There were also no significant differences between the first and second year results for these biomarkers (p>0.05). None of these parameters were correlated with hemoglobin A1c, body mass index and duration of T1DM. Interestingly, all parameters were negatively correlated to age of onset of T1DM (p<0.05).

**Conclusion:**

Our findings suggest that urinary biomarkers of fibrosis do not show an increase in diabetic children without microalbuminuria. The results also indicate that the risk of early fibrosis may increase as age of onset of T1DM decreases.

**What is already known on this topic?**

It has been demonstrated that mesangial expansion in diabetic nephropathy begins before microalbuminuria occurs. Only a few studies have reported alterations in urine levels of matrix metalloproteinases and tissue inhibitor of metalloproteinases in patients with type 1 diabetes mellitus and these studies have conflicting results.

**What this study adds?**

The indicators of fibrosis in urine do not increase in the early stage of type 1 diabetes mellitus. This finding suggests that the chronic changes in the kidney evolve at a later stage of the condition.

## Introduction

Type 1 diabetes mellitus (T1DM) is one of the most common chronic diseases of childhood (1,2). T1DM causes many macro- and microvascular complications. Diabetic nephropathy (DN) is one of the microvascular complications of T1DM (3,4). If T1DM is not well controlled, it eventually leads to end-stage renal disease (ESRD) due to renal fibrosis (5,6,7). It is known that increased production and decreased degradation of matrix leads to excessive accumulation of extracellular matrix (ECM) components and consequently to development of renal fibrosis (8). Matrix components are regulated by matrix metalloproteinases (MMPs) such as MMP2, MMP9 (9). They cleave denatured collagens, laminin and some cell adhesion molecules and growth factors such as transforming growth factor-β (TGF-β). Tissue inhibitors of metalloproteinases (TIMPs) are known as regulators of MMPs. TIMPs are usually inhibitory, although sometimes stimulate, MMP activity (10).

The prominent characteristic of DN is ECM accumulation and consequent development of mesangial expansion (8). These changes begin in the second stage of DN and become more prominent in later stages (11). Since MMPs regulate remodeling of ECM, they are important for tissue development (9). MMP2 and MMP9 have a crucial role on the degradation and regulation of ECM in the glomeruli (8). Therefore, MMPs may be involved in the pathophysiology of DN (8). TGF-β1 is an important growth factor also involved in kidney fibrosis and DN, via a number of pathways.

It has been suggested that DN usually manifests in adulthood and microalbuminuria is considered as the first laboratory sign of nephropathy (11). Usually, microalbuminuria occurs 6-15 years after diagnosis of T1DM. It would be clinically useful to identify earlier biomarkers than urinary microalbumin for predicting DN thus allowing more effective management and possibly delaying or preventing ESRD.

We hypothesized that the biomarkers of renal fibrosis may increase before microalbuminuria becomes manifest, since microalbuminuria is not the first finding of the disease, but a result of ongoing renal damage in DN (11). The aim of this study was to determine whether urine levels of MMP2, MMP9, TIMP1, TIMP2 and TGF-β1 increase in children with T1DM and serve to predict a progressive renal injury.

## Methods

Thirty-three consecutive patients (18 male, 15 female) with T1DM who attended the outpatient clinic of the Pediatric Endocrinology Department of İstanbul University Faculty of Medicine were enrolled in the study. Demographic and clinical characteristics of the patients are given in Table 1. To our knowledge, there are no standard normative data for urine levels of MMP2, MMP9, TIMP1, TIMP2, TGF-β1 in children by age group. For this reason, 24 healthy children (15 male, 9 female) were enrolled in the study as a control group. This study was approved by the İstanbul University of Local Ethics Committee (No: 2013/108) and written informed consent was obtained from the childrens’ parents.

A standard physical examination was performed in all patients and blood samples were drawn for biochemical examination. Height and weight measurements of the patients were taken by the same auxologist according to standard methods. Body mass index (BMI) in kg/m2 was evaluated according to the percentile curves of Turkish children and patients with a BMI above 95th percentile were considered obese (12). Standard deviation (SD) score (SDS) of BMI was calculated according to national data (12). Hypertension was defined as a systolic and/or diastolic blood pressure higher than the 95th percentile for age and gender (13).

Hemoglobin A1c (HbA1c) levels collected within the previous three months were collected from the patient files. Estimated glomerular filtration rate (GFR) values were calculated by using the Schwartz formula (14). Urinary assessment and urine culture were performed to exclude urinary tract infection for each patient. None of the patients had urinary tract infection. In addition no patient had a record of urinary tract infection, urolithiasis or nephrotoxic drug usage in the past three months. Patients with a urine microalbumin to creatinine (uMA/Cr) ratio greater than 30 mg/g in at least two of the three urine specimens were considered microalbuminuric (15).

Urine samples were obtained to measure urine levels of MMP2, MMP9, TIMP1, TIMP2, TGF-β1, microalbumin and creatinine. The samples were centrifuged at 4 ºC for 15 minutes at 4,000 x g. Until analyzed, the supernatants were stored at -80 ºC. All processes were performed under uniform conditions in all children. The Abbott Architect c16000 (Illinois, USA) analyzer with original kits was used to measure uCr and uMA, with uMA expressed in mg/L and uMA/Cr expressed in mg/g. Urine levels of MMP2, MMP9, TIMP-1, TIMP-2, TGF-β1 were assessed by enzyme-linked immunosorbent assay (ELISA) technique. Urine MMP2, MMP9, TIMP1 and TIMP2 levels were analysed following the manufacturer’s instructions, using Human MMP2 ELISA Kit (Cat no: YHB1973Hu), Human MMP9 ELISA Kit (Cat no:YHB1982Hu), Human TIMP-1 ELISA Kit (Cat no: YHB3003Hu), Human TIMP-2 ELISA Kit (Cat no: YHB3004Hu) and Human TGF-β1 ELISA Kit (Cat no: YHB3051Hu) purchased from YH Biosearch Laboratory (Pudong District, Shanghail, China). The intra-assay and the inter-assay coefficients of variation for MMP2, MMP9, TIMP1, TIMP2 and TGF-β were <10% and <12%, respectively. MMP2 and TIMP2 levels were expressed as ng/mL, MMP9 and TGF-β1 levels as ng/L. TIMP1 levels were expressed as pg/mL. The results were adjusted per unit of urine/Cr. Results of TGF-β1/Cr, MMP2/Cr, MMP9/Cr and TIMP2/Cr were expressed as ng/mg, and TIMP1/Cr as pg/mg. The same measurements were repeated after one year to determine whether urine levels of these markers altered in diabetic children with time.

### Statistical Analysis

Statistical calculations were performed with IBM SPSS Statistics for Windows, Version 22.0. (IBM Inc., Armonk, NY, USA). Besides standard descriptive statistical calculations (mean, standard deviation, median and interquartile range), a t-test was employed in the comparison of two groups and in the assessment of first and second year values. Kruskal-Wallis test was used to compare subgroups of diabetic control and diabetes duration. Pearson correlation test was used in the correlations between variables. Statistical significance level was established at p<0.05.

## Results

Mean ± SD age was 11.73±3.82 (range 4.5-17.8) years in the T1DM group and 11.6±3.0 years in the controls. There was no statistical difference between the two groups regarding age and gender distribution (p>0.05). Mean ± SD follow-up duration was 40.6±25.5 (range 6.4-93.9) months. All patients were on intensive insulin treatment. Mean ± SD BMI of the patients was 19.32±3.49 (range 13.72-26.65) and mean ± SD BMI SDS was 0.08±1.01 (-1.15-2.32). Normal blood pressure was observed in all patients. Mean ± SD estimated GFR was 157.46±34.61 mL/min/1.73 m2 (range 107.25-303.32). Mean ± SD HbA1c was 9.11±2.17% (range 5.7-15.5). Mean ± SD uMA/Cr was 20.17±47.51 (range 1.28-239.41) mg/g Cr. Urine MMP2/Cr, MMP9/Cr, TIMP1/Cr, TIMP2/Cr, TGF-β1/Cr were not different in the patient and control groups (p>0.05) (Table 2). There was also no significant difference between the results of the first and second year samples of the diabetes patients in these biomarkers (p>0.05). None of these parameters were correlated to age, HbA1c, BMI and duration of T1DM. Interestingly, all parameters were negatively correlated to the age of onset of T1DM (p<0.05) (Table 3). A positive correlation was found among urine MMP2/Cr, MMP9/Cr, TIMP1/Cr, TIMP2/Cr and TGF-β1/Cr (p<0.05). Microalbuminuria was present in only three patients. Among the three patients with microalbuminuria, only one had higher values of the urine biomarkers than the patients group mean values.

The patients were divided into two subgroups according to duration of diabetes: 0-5 years (n=19) and over 5 years (n=14). There was no difference between the two groups according to urine MMP2/Cr, MMP9/Cr, TIMP1/Cr, TIMP2/Cr, TGF-β1/Cr values (Table 4). Also, the patients were divided into three groups depending on diabetic control as measured by HbA1c: good (n=8), moderate (n=14) and poor glycemic control (n=11) (see Table 5). The urine biomarkers did not differ between the groups with good, moderate or poor glycemic control (Table 5).

## Discussion

Since changes in the ECM are a significant pathogenetic mechanism in DN, we hypothesized that the onset of alterations in urine MMP2, MMP9 and TIMP1, TIMP2 may occur prior to appearance of microalbuminuria. We also expected this change in markers of renal fibrosis to become more prominent with time because kidney injury in DN is a progressive process. However, our results did not support our hypothesis. Urine MMP2/Cr, MMP9/Cr, TIMP1/Cr, TIMP2/Cr values were essentially similar in the patients and controls, and they did not change over one year in the T1DM patients. From these results it seems that chronic changes in DN do not begin in the early stages of the disease.

The role of MMPs in the pathogenesis of DN is not fully understood. Although it has been demonstrated that dysregulation of MMPs occurs in DN, the reported results are contradictory (8). Decreased expression of MMP2 and MMP9 was reported in several experimental studies of DN, while other studies reported increased expression of MMPs (9,16,17). Additionally, it has been noted that while MMP2 knock-out mice show an exacerbation of DN, MMP9 knock-out mice show an attenuation of DN (18,19). Expression of TIMP1 and TIPM2 are increased in DN (8,9,20,21).

There are only a few studies evaluating urinary MMPs in patients with diabetes. McKittrick et al (22) evaluated urine activities of MMP2 and MMP9 in the urine of patients with T1DM and they found that urinary MMP9 did not differ between the patients and controls; our results are in concordance with these earlier findings. However, unlike our results, they reported an increase in the activities of MMP2 (22). Lauhio et al (23) demonstrated elevation of the urinary activity of MMP9 in adult patients with type 2 DM. However, their study group was quite different from our group. Most of their patients had macroalbuminuria and a diabetes duration longer than 10 years. Tashiro et al (24) evaluated urinary MMP9 in adult patients with type 2 DM who are different our study population. They did not find any differences in urinary MMP9 between normo/microalbuminuric patients and healthy controls, findings similar to our results. However, in this study, urinary MMP9 were found to be higher in macroalbuminuric patients. van der Zijl et al (25) evaluated urinary MMP2 and MMP9 levels in adult patients with type 2 DM and reported that urinary MMP9 levels were higher in the microalbuminuric group than in the controls, while there was no difference in MMP2 activity. Elevation of urinary MMP9 activity was found to be related to older age, longer duration of diabetes, high levels of HbA1c and increased blood pressure. Thrailkill et al (26) evaluated MMP2 in T1DM patients and found that MMP2 increased in the plasma and urine although they did not find any differences between patients and controls in TIMP1 and TIMP2 concentrations. Similarly to our results, when they evaluated the younger groups (<18 years) they did not find any differences according to urine MMP2/Cr and total urine MMP2 concentrations (26). In a later study from the same group Thrailkill et al (27) reported elevation of urinary MMP9 in normoalbuminuric patients with T1DM with duration of diabetes being nine years, a disease duration longer than that of our study group. These studies suggest that the role of the clinical use of urinary MMPs is not fully understood. These differences between the studies may be due to the fact that the patient groups as well as the evaluation method of urine MMPs are quite different from one another. According to these studies, diabetes duration has a significant role on the alteration of urinary MMP2 and MMP9. Also this alteration appears to become more prominent in the later stages of DN. The mean duration of diabetes was only 3.5 years in our patients. ECM accumulation and mesangial expansion begin in the second stage of DN (11). Also, with the exception of three patients, our patients did not have microalbuminuria. We could not demonstrate any differences according to these biomarkers, probably because of the short duration of the diabetic state and because our patients had not yet reached the second and/or later stages of DN. Based on a few previous studies which demonstrated higher values of urine MMP2/Cr and MMP9/Cr in adult diabetic patients, we thought that these markers may increase with time as diabetic injury progresses (24,25,26,27). We also did not find any difference in the values of urinary MMP2/Cr, MMP9/Cr, TIMP/Cr and TIMP2/Cr at initial measurement and when measured a year later. These results show that urine levels of these markers do not change in the early phases of DN and cannot predict early progression of DN.

Some comorbid conditions other than diabetes mellitus such as renal scars, nephrotic syndrome, focal segmental glomerulosclerosis, pancreatic cancer and chronic kidney failure may also affect urine MMP2 and MMP9, TIMP1 and TIMP2 and TGF-β1 concentrations (28,29,30,31,32,33). However, our diabetic patients did not have any known comorbid disorders.

TGF-β1 is considered as the most important cytokine in glomerular and tubulointerstitial fibrosis (34). Additionally, expression of TGF-β1 is increased with hyperglycemia, thus TGF-β1 is involved in various pathways having a role in the pathogenesis of DN (34). Furthermore, MMPs not only cleave ECM proteins but also target some non-ECM proteins, including TGF-β1, and activation of the TGF-β/Smad signal pathway which is accompanied by MMP2 and MMP9 upregulation (9,10). Therefore, in addition to urinary MMPs, we evaluated urinary TGF-β1 in our patients. Again TGF-β1 was not increased in our patients. In fact, this result was consistent with our results for MMP2 and MMP9. These results suggest that chronic fibrotic changes may not become apparent and these markers do not increase in the urine in the early phases of diabetic kidney injury.

Poor metabolic control, higher BMI, longer duration of disease and onset of diabetes at puberty have been identified as risk factors for DN. Therefore, we evaluated the correlations between these biomarkers and HbA1c, BMI, duration of T1DM and age of onset of T1DM. Only age of onset was negatively correlated with all these biomarkers of renal fibrosis. This finding suggested that among the indicators of poor prognosis of T1DM in terms of renal damage, the most important determinant seems to be the age of onset of the diabetic state.

### Study Limitations

The limitations of our study are the relatively small sample size with only three microalbuminuric patients. Thus, we were not able to compare microalbuminuric and normoalbuminuric patients for these markers. We did not perform kidney biopsies and thus we are not in a position to make any statements on the pathological DN stage of our patients. Despite these limitations our study has yielded important results. One of the most significant findings was that there were no difference between patients and controls according to these biomarkers and this finding did not change after one year of follow-up. These findings weaken the role of these biomarkers in the detection of early diabetic kidney injury. In this respect, future studies with longer follow-up and larges samples in a pediatric age group are needed to highlight this issue.

## Conclusion

In conclusion, our findings suggest that urinary biomarkers of fibrosis are not increased in diabetic children without microalbuminuria even when disease duration is longer than five years.

## Figures and Tables

**Table 1:**
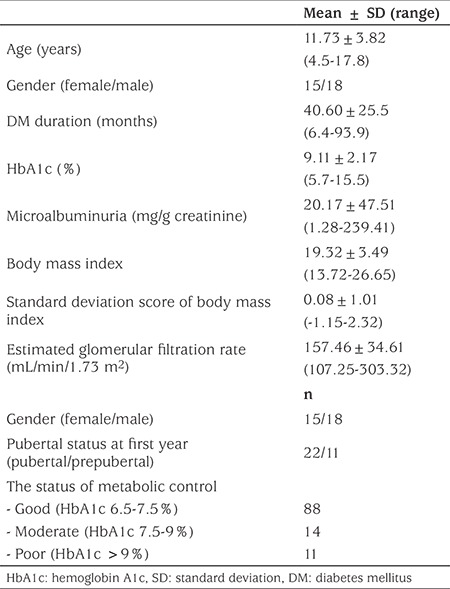
Demographic and clinical characteristics of the patients

**Table 2:**
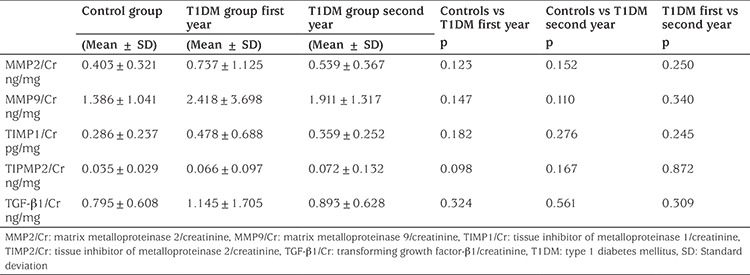
Urinary biomarkers in the patients in the first and second years of onset vs the controls

**Table 3:**
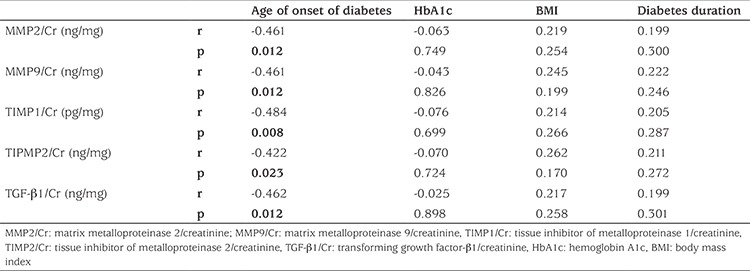
Correlations of urine matrix metalloproteinase/creatinine and tissue inhibitor of metalloproteinases/creatinine with age of onset of the diabetes, with hemoglobin A1c, body mass index and diabetes duration

**Table 4:**
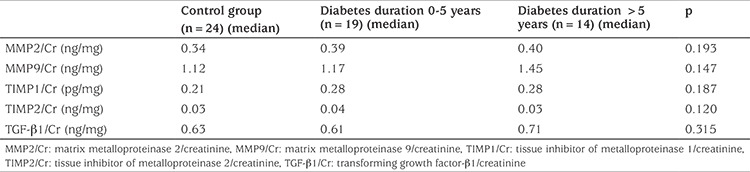
The relationships between urine biomarkers and diabetes duration

**Table 5:**
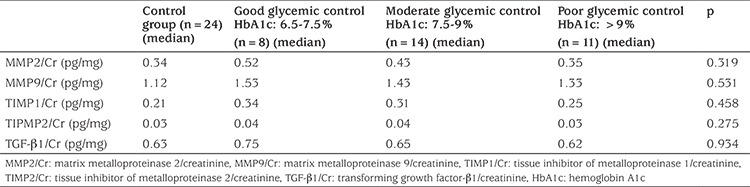
The relationships between urine biomarkers and diabetic control
